# Effects of Bromination-Dehydrobromination on the Microstructure of Isotropic Pitch Precursors for Carbon Fibers

**DOI:** 10.3390/polym12123059

**Published:** 2020-12-20

**Authors:** Dingcheng Liang, Deqian Liu, Shuai Yang, Changyu Lu, Qiang Xie, Jinchang Liu

**Affiliations:** School of Chemical and Environmental Engineering, China University of Mining and Technology (Beijing), Beijing 100083, China; liangdc@cumtb.edu.cn (D.L.); ldq_cumtb@163.com (D.L.); iqshuai@163.com (S.Y.); Squidward@student.cumtb.edu.cn (C.L.); dr-xieq@cumtb.edu.cn (Q.X.)

**Keywords:** carbon fiber, isotropic pitch, bromination-dehydrobromination, microstructure

## Abstract

In this work, isotropic pitch precursors are synthesized by the bromination-debromination method with ethylene bottom oil (EO) as the raw material and bromine as the initiator for pitch formation and condensation reactions. The aggregation structure, molecular weight distribution, and molecular structure of isotropic pitch precursors are characterized by thermal mechanical analyzer (TMA), MALDI TOF-MS, and ^13^C NMR, respectively, for revealing the mechanism of synthesis of isotropic pitch precursors. The results show that at low bromine concentrations, polycyclic aromatic hydrocarbons (PAHs) were mainly ordered in cross-linked structures by bromination-debromination through substitution reactions of side chains. The condensed reactivity can be improved by the effect of bromine, meaning that condensation reaction was aggravated by the method of bromination-dehydrobromination. In the presence of excess bromine, the cross-linked stereo structure of PAHs changed to the planar structure of condensed PAHs, which was not conducive to the subsequent spinning and preparation of carbon fibers.

## 1. Introduction

Carbon fiber is an important material that is widely used in the automobile, aerospace, and sport industries, as well as other industries [1,2]. General-purpose carbon fibers (GPCFs) that are prepared with isotropic pitch as a precursor have the advantages of a low cost and appropriate mechanical properties. Therefore, compared with high-performance PAN (polyacrylonitrile)-based carbon fibers and mesophase pitch-based carbon fibers, GPCFs have better application potential [3,4,5,6]. The properties of GPCFs are significantly affected by the qualities of raw materials and the properties of isotropic pitch precursors [7]. Presently, raw materials used for the preparation of GPCFs mainly come from the byproducts of coal and petroleum processing, such as coal tar, anthracene oil, ethylene bottom oil (EO), and so forth to realize the low-cost preparation of GPCFs [8,9,10,11].

EO is a byproduct in the process of producing ethylene from petroleum hydrocarbons by high-temperature pyrolysis. Affected by different raw materials and processes, the yield of EO varies by approximately 15%~20% of ethylene production. With a high carbon content and low ash content, EO is a cheap material that can be used for the potential preparation of isotropic pitch and carbon fiber materials. Starting from EO, the preparation methods of isotropic pitch precursors and GPCFs include thermal condensation [12,13], co-carbonization with PVC [14,15], and bromination-debromination [16]. However, a higher temperature is required to synthesize pitch precursors by thermal condensation and co-carbonization with PVC. Most of the light components in EO easily evaporate, resulting in a low yield of isotropic pitch. The spinnability of pitch precursors and the mechanical performance of final carbon fibers are largely determined by the molecular structures of isotropic pitch precursors. EO can be used to prepare isotropic pitch precursors with excellent spinnability. Additionally, the high reactivity characteristic also causes excessive polycondensation, resulting in the formation of insoluble coke and carbon particles, which affect the spinning performance. To improve the yield and prepare pitch precursors with good spinning performance, Ge et al. [17] and Kim et al. [18] proposed a method for preparing high quality pitch precursors with methyl naphthalene as the material by bromination-debromination. The polymer pitch consisted of numerous repeating monomers of methylene-bridged naphthalene rings with linear structures. Bromination-debromination is a process in which a hydrogen atom on the aliphatic side chain of an aromatic ring is replaced by a bromine radical, and then the bromine is removed in the form of hydrogen bromide at approximately 300 °C. Through this process, aromatic compounds are neatly connected through side chain bridge bonds [19,20]. Bromine can form bromine free radicals under light or low temperature conditions (110 °C). Presently, there are few studies on the preparation and performance optimization of isotropic pitch-based carbon fibers by bromination-debromination. However, for complex EOs, the reaction mechanism of bromination and debromination for preparing pitch precursors requires further study.

The microstructure of pitch precursor plays a significant role in properties and mechanical performance of obtained carbon fibers. Molecular structure regulation in the process of synthesis of pitch precursor has attracted more attention, and the development and application of bromination-dehydrobromination and chlorination-dehydrochlorination methods prove it very well [14,15,16]. The molecular structure of isotropic pitch precursors is mainly composed of condensed aromatic hydrocarbons that are thought to be aggregate structures in the pitch microstructure, and the aggregate structure is surrounded by aliphatic groups [1]. The structural relationship between condensed aromatic hydrocarbons and surrounding aliphatic groups determines the properties of pitch precursors as well as affects the mechanical performance of carbon fibers. The target of molecular structure regulation for pitch precursor synthesis is to form more linear and naphthenic structure, so as to obtain excellent spinnable and mechanical performances. However, the previous work mainly focused on improving properties of pitch precursors by optimizing reaction conditions of bromination, rather than regulating the molecular structure [14,16]. Thermomechanical analysis is widely used to analyze the thermodynamic properties and aggregate structure of fiber materials [21,22], which can determine the glass transition and softening temperatures of pitch- and pitch-based carbon fibers. In this study, isotropic pitch precursors are synthesized by the bromination-debromination method with EO as the raw material and bromine as the additive. The thermal mechanical analyzer (TMA) is used to study the synthesis process and aggregate structure of isotropic pitch precursors. The potential use of the bromination-debromination process is further explained in this study and verified by MALDI TOF-MS and ^13^C NMR.

## 2. Materials and Methods

### 2.1. Materials and Preparation of Isotropic Pitch Precursors

Ethylene bottom oil, which was provided by China Petrochemical Corporation, was utilized to prepare the isotropic pitch precursors. Bromine was purchased from Shanghai Chemical Co., Ltd. in analytical purity.

**Figure 1:**
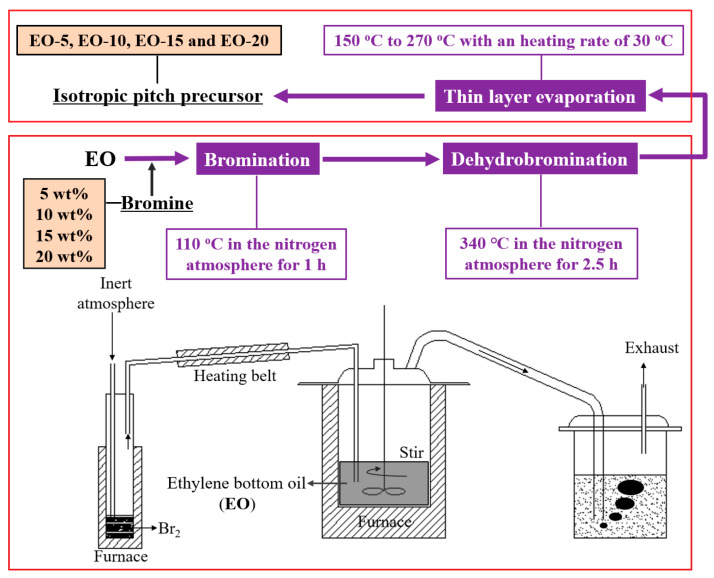
The preparation process of isotropic pitch precursors by bromination-dehydrobromination.

The preparation process for isotropic pitch precursors is shown in Figure 1. Approximately 100 g of EO was added to a glass reactor and heated slowly to 110 °C, with a heating rate of 5 °C/min under a nitrogen atmosphere. To ensure that EO was completely melted, the sample was sustained at 110 °C for at least 10 min. Then, through nitrogen transportation, bromine was added with mass fractions of 5 wt %, 10 wt %, 15 wt %, and 20 wt %. The reaction was conducted at 110 °C for 1 h with a nitrogen flow rate of 200 mL/min and a stirring speed of 100 rpm. After bromination, the reactor was heated to 340 °C with a heating rate of 5 °C/min and then sustained at this temperature for 2.5 h to allow for dehydrobromination to occur. The products after bromination-dehydrobromination were the initial isotropic pitches. For improved melt spinning, it was necessary to remove the residual bromine and some of the light components from the bromination-dehydrobromination products. Therefore, the products after bromination-dehydrobromination were placed in an evaporator for thin-layer evaporation (TLE). The temperature of the evaporator was heated from the initial 150 to 270 °C with a heating rate of 30 °C/min. The bromination-dehydrobromination products were continuously evaporated by thin-layer evaporation until the softening point of each sample was increased to 240 °C. The resulting samples were the final spinnable isotropic pitch precursors. On the basis of the amount of bromine added, the samples of isotropic pitch precursors were labelled EO-5, EO-10, EO-15, and EO-20.

### 2.2. Characterization of Isotropic Pitch Precursors

The carbon and hydrogen contents of isotropic pitch precursors were determined by an element analyzer (Vario MACR, Elementar, Langenselbold, Germany). The softening point of the isotropic pitch precursors was measured by the hot plate method.

**Figure 2:**
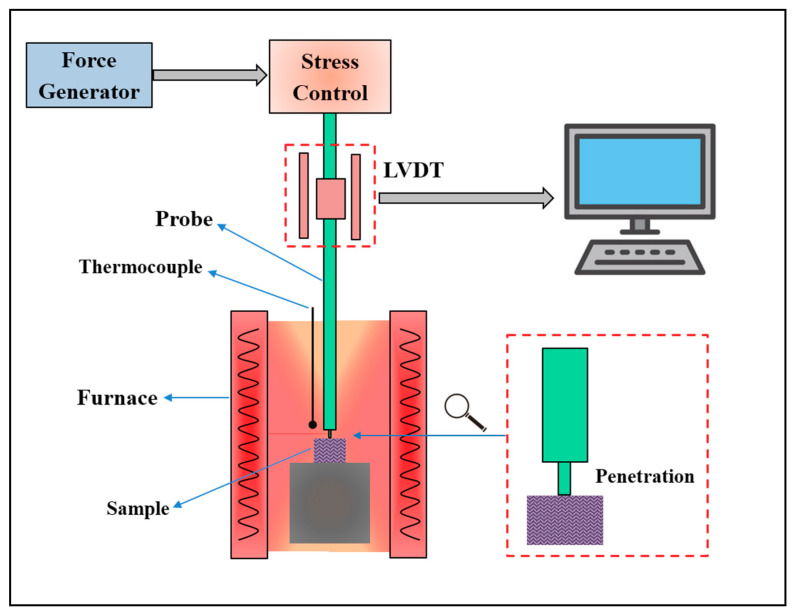
Schematic diagram of thermal mechanical analyzer (TMA) for isotropic pitch precursor characterization.

The thermal properties of isotropic pitch precursors were characterized by a thermal mechanical analyzer (TMA/SS7300, Seiko Co. Ltd., Tokyo, Japan). The structural schematic diagram of TMA is shown in Figure 2. After grinding, the sample was placed into a corundum crucible and calcined at 270 °C for 30 min to melt the sample. Then, the calcination samples underwent TMA. Measurements were collected at a constant load of 49 mN, an increased temperature from 25 to 350 °C, and a heating rate of 5 °C/min under nitrogen flow of 100 mL/min. Before the formal test, the baseline calibration was completed with a quartz probe to test the blank corundum crucible under the same test conditions. This was conducted to avoid the influence of quartz probe deformation on the test results.

The molecular weight distributions of the isotropic pitch precursors were measured by MALDI TOF-MS (Autoflex III, Bruker Co. Ltd., Karlsruhe, Germany). The samples were dissolved in high-purity tetrahydrofuran (Shanghai Chemical Co., Ltd., Shanghai, China) without a stabilizer. The sample concentration was 0.8% *w*/*v* and the molecular weight was measured in the mass range of 100–1000 *m*/*z*. The monomer, dimer, trimer, and tetramer contents in the isotropic pitch precursors were calculated by integrating MALDI TOF-MS data.

The position of the carbon atoms in the isotropic pitch precursors was determined by ^13^C solid-state nuclear magnetic resonance spectroscopy (^13^C NMR, Avance III 400, Bruker Co. Ltd., Karlsruhe, Germany). The rotation speed of the rotor was 8 kHz and the collection time was more than 7 h.

## 3. Results and Discussion

### 3.1. Basic Properties of Pitch Precursors

EO is mainly composed of complex polycyclic aromatic hydrocarbons (PAHs) with abundant side chain groups. The product of EO by thermal condensation polymerization, which requires high temperature and pressure, has a low degree of polymerization and low yield [13]. Moreover, the pitch-based carbon fibers prepared by these isotropic pitch precursors are of poor quality. In addition, under the condition of direct thermal polycondensation, most of the light components in EO are released in the form of gas before the polymerization and condensation reactions, resulting in a low product yield. It is well known that the aliphatic side chains of aromatic rings are prone to radical substitution of halogens under visible light or thermal conditions, and some researchers have proposed the preparation of isotropic asphalt precursors by the bromination-dehydrobromination method [19]. The yield, the softening point, and C and H contents of the isotropic pitch precursor prepared by bromination-dehydrobromination are shown in Table 1. These results show that as the bromine addition increased, the yield of basic pitch derived from bromination-dehydrobromination increased, but decreased slightly until the addition of bromine was 20%. In the bromination reaction at 110 °C, the hydrogen atom on the aliphatic substituents of aromatic compounds was easily replaced by bromine radicals. Then, the bromine atom was removed in the form of hydrogen bromide in the dehydrobromination reaction at 340 °C, and the oligomerization of these aromatic compounds resulted in the retention of some lighter components in the EO. With increasing bromine content, more of the light components were connected with aromatic compounds through bromine substitution reactions and dehydrobromination reactions, resulting in an improved basic pitch yield. However, the possible reasons for the decrease in the basic pitch yield of EO-20 are discussed in the following sections.

The softening point of the isotropic pitch precursor affects the operation temperature of melt spinning. Therefore, it was necessary to complete a thin layer evaporation heat treatment on the basic pitch to remove residual bromine and some of the lighter components. With this treatment, the isotropic pitch precursor was produced, and the yield of the isotropic pitch precursor continued to decline, which was related to the production economy. The results from Table 1 show that the yield of the isotropic pitch increased as the bromine addition increased, and the yields of the isotropic pitch by EO-5, EO-10, EO-15, and EO-20 were 41.8%, 45.2%, 51.1%, and 58.4%, respectively. This suggests that as more bromine was added, there were more residual bromine and light components present in the basic pitch. Some interesting results were also obtained by observing the variation in the C and H contents of the isotropic pitch precursors from Table 1. As the addition of bromine to the EO increased from 5% to 20%, the C content in the isotropic pitch precursor prepared by bromination-dehydrobromination increased, while the H content decreased. The ratio of C to H increased with the addition of bromine, and the unsaturation of the isotropic pitch precursor increased. The results indicated that, not only did oligomerization reactions occur, but condensation reactions also occurred in the process of bromination-dehydrobromination. The more bromine that was added, the more bromine was removed, which led to more condensation reactions and higher aromaticity of the isotropic pitch precursor. Hence, it was inferred that the removal of bromine in EO-20 resulted in the condensation polymerization of aromatic compounds to form PAHs, while the oligomerization of aromatic compounds with small molecules occurred less often. Therefore, the small molecules were released from EO-20, which resulted in a decrease in the yield of isotropic pitch precursors.

### 3.2. Thermal Mechanical Analysis

**Figure 3:**
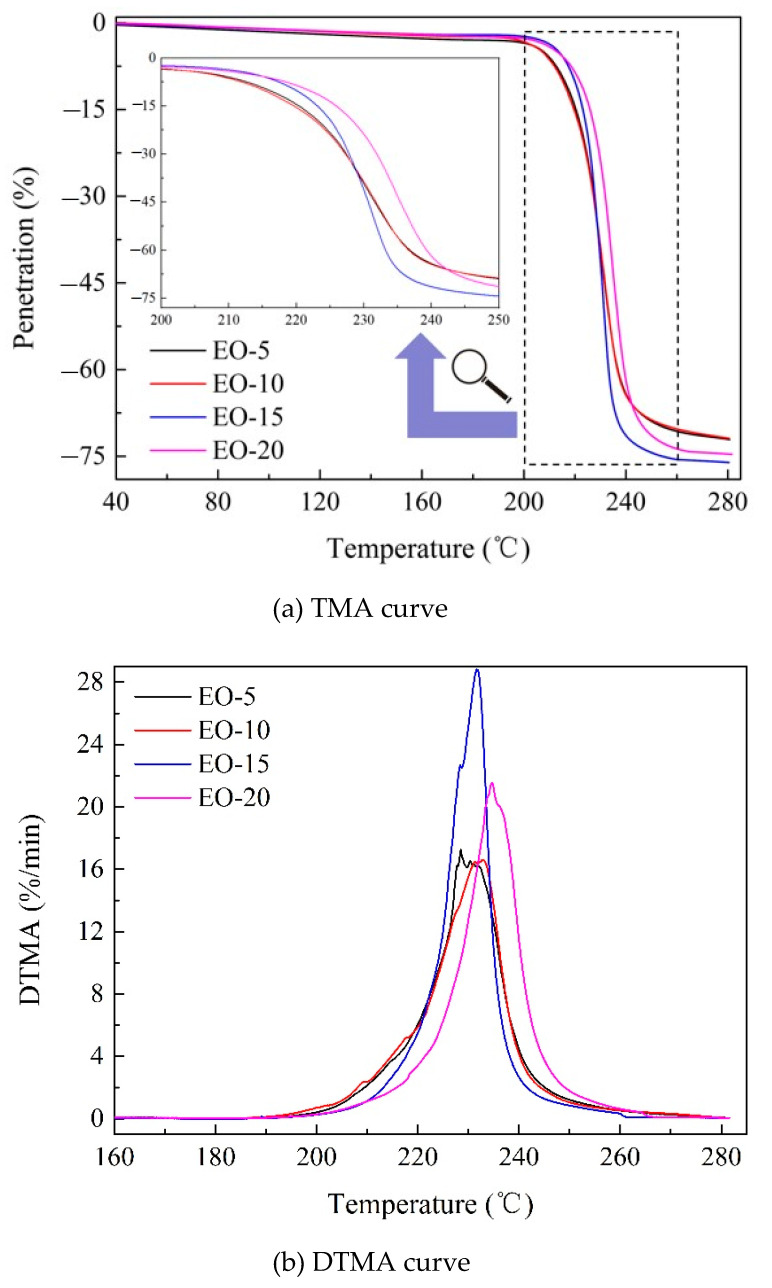
Thermal properties of isotropic pitch precursors characterized by thermomechanical analysis, (**a**) TMA curve and (**b**) DTMA curve.

To further realize the aggregation structure of the isotropic pitch precursor, thermal mechanical analysis was performed and the results are shown in Figure 3. Under the driving force of an external load, the probe always exerted constant stress on the isotropic pitch surface, which was 48 mN. With increasing temperature, the isotropic pitch precursor started to soften, and the temperature was approximately 190 °C, which was an indication that the molecular chain segment began to move. Meanwhile, the driving force of the probe was greater than the resistance of the isotropic pitch precursor, and the displacement and speed of penetration increased continuously. When the temperature increased further, the resistance of the probe was greater than 48 mN, the probe displacement increased, while the penetration speed decreased and returned to 0, and the temperature was 280 °C. Hence, the temperature range for deformation of the isotropic pitch precursors under the action of probe penetration was approximately 190 to 280 °C. 

For the isotropic pitch precursors with PAHs as the main structure, the difficulty of probe penetration into the isotropic pitch precursor was closely related to the size, arrangement, and cross-linking degree of their molecular chains. From Figure 3a, the maximum penetration of EO-5, EO-10, EO-15, and EO-20 was 72.10%, 71.88%, 76.09%, and 74.68%, respectively. Figure 3b reflects the maximum speed of probe penetration. According to Figure 3a,b, the difficulty order of probe penetration was EO-10 > EO-5 > EO-20 > EO-15. Specifically, TMA probe penetration into the precursor was difficult at first, then became easy, and finally became difficult again as the bromine addition increased. The probable reason for this phenomenon was that bromination-dehydrobromination with different bromine contents changed the molecular structure of the EO, which was reflected by the variation in the macroscopic physical properties of the EO. In the presence of bromine free radicals, the H in the aliphatic side chain of the aromatic ring was easily replaced by these free radicals; then, bromine in the side chain of the aromatic ring was easily removed after combining with hydrogen on the other aromatic ring at high temperature, which led to the oligomerization of aromatic rings through cross-linking of aliphatic side chains. When the quantity of bromine was small, the side chain of the aromatic ring was mono-substituted by bromine. Through the removal of hydrogen bromide, the aliphatic side chains of the aromatic ring formed a cross-linking structure, which resulted in the formation of polymers with a low degree of polymerization. As the molecular structure of these isotropic pitch precursors was three-dimensional, the polycondensation of their molecular chains became orderly after high temperature softening, which resulted in the influence of space resistance on the penetration of the probe, a large resistance and a small penetration. With the increase in bromine, the side chains of the aromatic rings underwent multiple substitutions by bromine, which led to further deepening of the degree of cross-linking and polymerization; thus, probe penetration became more difficult when the precursors were derived from EO-10 due to softening at high temperature. However, excessive bromine addition caused cyclization and aromatization between aliphatic side chains and aromatic rings, which resulted in an increase in the condensation degree of the precursor. The ratio of C/H shown in Table 1 increased as bromine addition increased, which also supports this inference. As the condensation product, the pitch precursor with fused aromatics was transformed by graphitization and the intermolecular force decreased; therefore, probe penetration became easy after the precursor, derived from EO-15, was softened at high temperature. When the bromine content of the EO increased to 20%, the condensation degree of the aromatic rings in the precursor was high, and the molecular size of the fused aromatics was large. The large steric hindrance caused by the greater disorderly arrangement of the molecules resulted in the difficulty of probe penetration after precursor softening at high temperature.

Through the above analysis, this study found that the process of probe penetration into the isotropic pitch precursor was closely related to the molecular weight and flexibility of the molecular chain. Figure 3b shows that the probe penetration speeds of the isotropic pitch precursors derived from EO-15 and EO-20 were slower than those of EO-5 and EO-10; this is consistent with the fact that the precursors derived from EO-15 and EO-20 had a higher condensation degree and worse flexibility of the molecular structure. Moreover, due to the highest degree of aromatic ring condensation of precursors derived from EO-20, the highest temperature was required to excite the molecular chain from the freezing state to the moving state; thus, the penetration speed was the slowest at the beginning of precursor softening, and its DTMA curve shifted to the right. Due to the large molecular size and steric hindrance caused by the molecular structure, probe penetration was more difficult than that derived from EO-15, but was easier than that derived from EO-5 and EO-10 with a large number of cross-linking structures.

**Figure 4:**
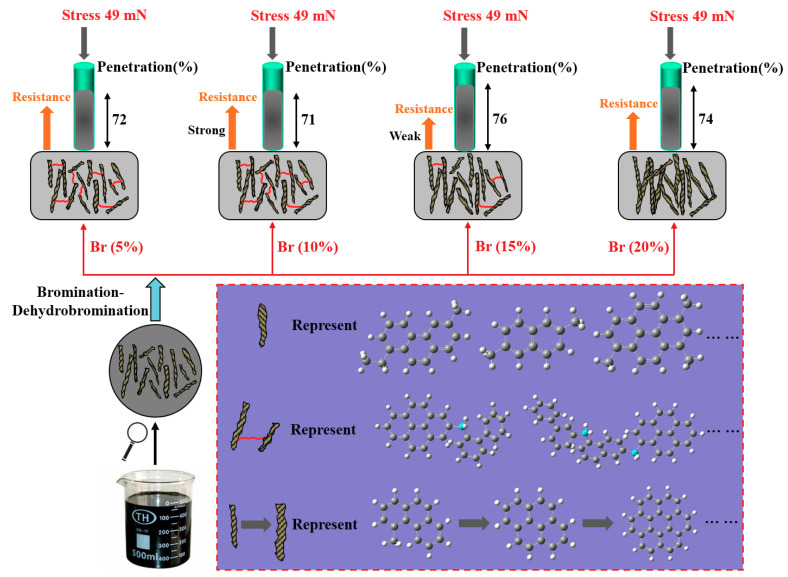
Effects of bromination-dehydrobromination on the microstructure of isotropic pitch precursors.

In conclusion, the preparation process of isotropic pitch precursors derived from EO by bromination-dehydrobromination mainly involved oligomerization and condensation reactions through aromatic ring side chain cross-linking. When the amount of bromine was low, oligomerization was the main reaction. The degree of condensation increased as the bromine addition increased, and the effect of bromination-dehydrobromination on the microstructure of the isotropic pitch precursor derived from the EO is illustrated in Figure 4.

### 3.3. Molecular Weight Distributions

**Figure 5:**
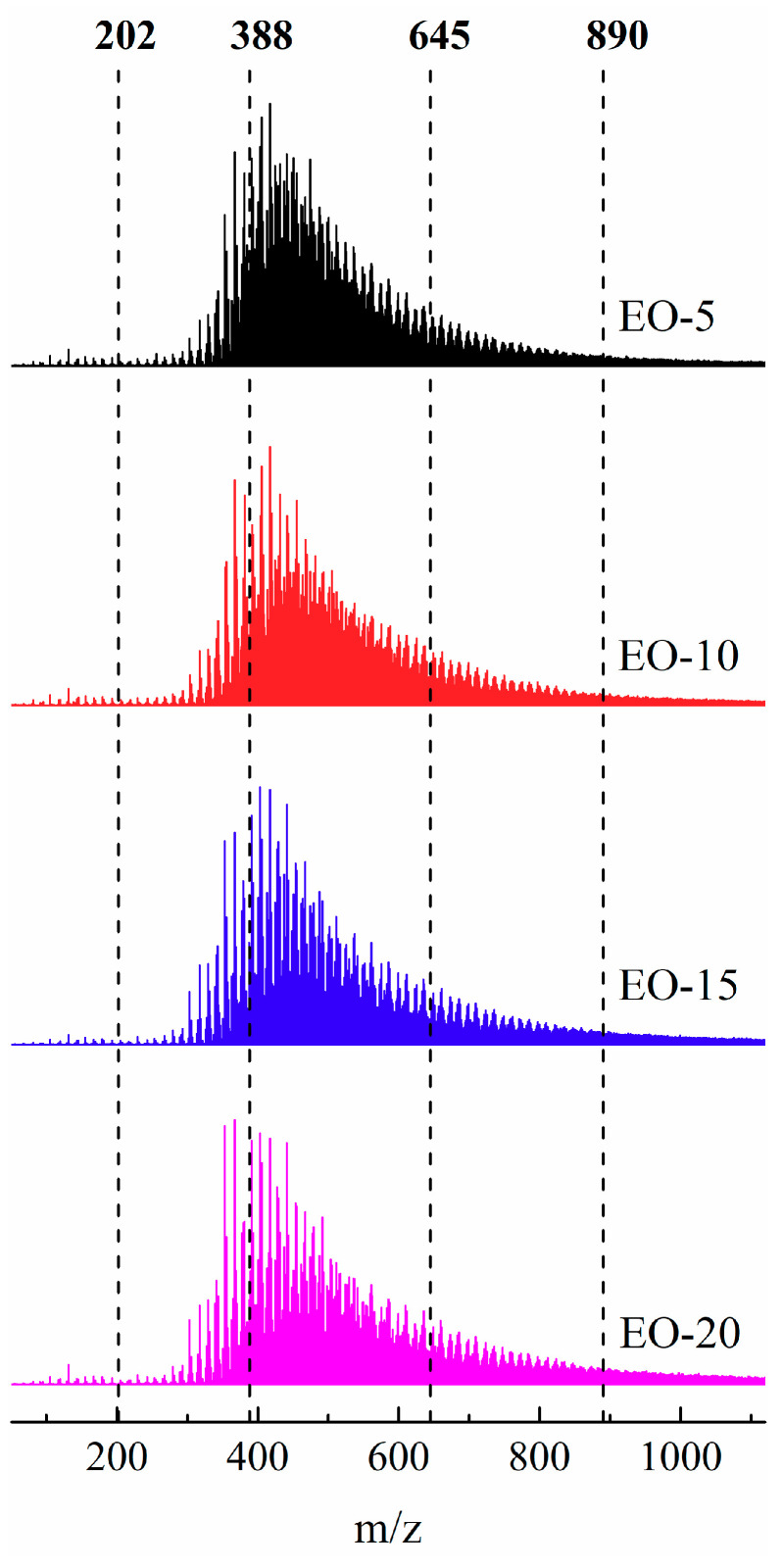
The molecular weight distribution of isotropic pitch precursors by MALDI TOF-MS.

To further explore the bromination-dehydrobromination process, the molecular weight distributions of isotropic pitch precursors prepared from EO by the bromination-dehydrobromination method were characterized by MALDI TOF-MS with a determining range of 100 to 1000 *m*/*z*, and the results are shown in Figure 5. Specifically, the molecular weight of the small molecules is less than 202 *m*/*z*, and the ranges of monomers, dimers, trimers, and tetramers are 202–388 *m*/*z*, 388–645 *m*/*z*, 645–890 *m*/*z* and 890–1120 *m*/*z*, respectively [19,23,24]. There are few small molecules in the isotropic pitch precursors based on Figure 5, which is consistent with the light components in EO that were released or cross-linked with the aliphatic side chains of the aromatic ring during the bromination-dehydrobromination or thin-layer evaporation process. The molecular weight of the isotropic pitch precursor was mainly distributed at 388–645 *m*/*z*, meaning it was mainly composed of dimers; the signals of monomers, trimers, and tetramers were also detected by MALDI TOF-MS. In particular, the presence of trimers and tetramers indicates that the oligomerization of aromatic compounds occurred in the preparation process of these isotropic pitch precursors. Therefore, it was necessary to further explore the molecular weight distributions of the isotropic pitch precursors, which can clarify the effect of bromination-dehydrobromination on the microstructure of isotropic pitch precursors.

The composition and content of compounds with different molecular weights in the isotropic pitch precursors can be quantified using the integral calculation of the curves drawn by the MALDI TOF-MS results, which are summarized in Table 2. The average relative molecular weight of the isotropic pitch precursors increased as the bromine addition increased. Combined with the variation trend of the isotropic pitch precursor, yield shown in Table 1, it was inferred that the introduction and removal of bromine caused the oligomerization of light components with aromatic rings in EO, and the addition of bromine further increased the degree of aromatic polymerization, and condensation reactions even occurred. Additionally, more information regarding the content of dimers was found to be 39.87% to 54.37%, which were the main components of the precursors. The content of small molecules was 0.05% to 0.12%, which could be ignored. As the bromine content increased from 5% to 20%, the monomer and dimer contents decreased from 6.70% and 54.37% to 5.20% and 39.87%, respectively, while the trimer and tetramer contents increased from 25.58% and 12.94% to 33.45% and 21.43%, respectively. This demonstrated that the hydrogen on the side chain of aromatic rings was substituted by bromine, and the removal of hydrogen bromine caused the PAHs to form oligomers through side chains of aromatic rings by cross-linking in the process of bromination-dehydrobromination. The more bromine that was added, the more that hydrogen bromine was removed, and the more likely the cross-linking reaction occurred, creating a higher degree of polymerization and a greater molecular weight. This was consistent with the TMA analysis results of the isotropic asphalt precursors.

### 3.4. ^13^C NMR Analysis

**Figure 6:**
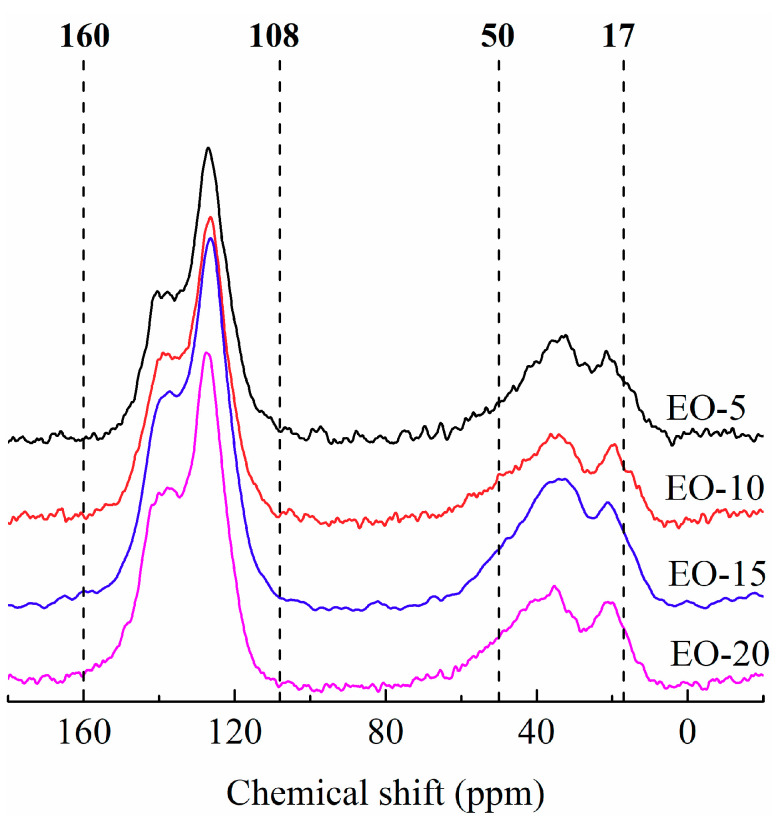
^13^C NMR spectra of isotropic pitch precursors.

In order to more accurately understand the evolution of EO molecular structure in the process of bromination-dehydrobromination, ^13^C NMR of isotropic pitch precursors was performed in this study, and the results are depicted in Figure 6. Previous research confirmed that the carbon atoms of aliphatic chains and aromatic rings were in the ranges of 17–50 ppm and 108–160 ppm in ^13^C NMR spectra, respectively [19,25]. Based on Figure 6, the carbon atom signal of the aromatic ring was stronger than that of the aliphatic chain, which suggested that the molecular structure of the isotropic pitch precursor was mainly composed of aromatic groups. This was also consistent with the fact that EO was mainly composed of side chain-rich PAHs. In the process of bromination-dehydrobromination, PAHs were cross-linked and polymerized by aliphatic side chains, which resulted in cross-linking bonds derived from the side chains of aromatic rings; meanwhile, aliphatic hydrocarbons were produced in the pyrolysis reaction.

Results of the carbon atom content at different positions were obtained by the deconvolution of the ^13^C NMR carbon spectrum, as shown in Table 3. Carbon atoms in the aliphatic chain can be subdivided into methyl carbon (CH_3_), methylene carbon in the side aliphatic chain (CH_2_), and methylene carbon in a position to two aromatic rings (CH_a2_); the chemical shifts of these carbons in the ^13^C NMR spectra were 17–23 ppm, 23–34 ppm and 34–50 ppm, respectively. Aromatic ring carbon atoms can be further divided into protonated aromatic ring carbon (CH_ar_), peri-condensed aromatic carbon (C_ar3_), aromatic carbon joined to aliphatic chains (C_sar_) and cata-condensed aromatic carbon with either heteroatomic or aromatic substituents (C_ar2_), with the chemical shift of 108–115 ppm, 115–129.5 ppm, 129.5–132.5 ppm and 132.5–160 ppm [19,25], respectively.

As can be clearly seen from the above table, the amount of CH_3_ and CH_ar_ decreased with increasing bromine addition. It is generally known that these CH_3_ mainly exist in the side chain of aromatic rings, which is the best position for a bromine radical substitution reaction combined with the variation of CH_ar_. Again, this suggests that the bromine on the side chain of aromatic rings was easily removed, with the hydrogen on the other aromatic ring in the form of hydrogen bromide at high temperature. Then, two PAHs were cross-linked through the side chains of aromatic rings, which was also confirmed by the high content of CH_a2_. Moreover, as the bromine addition increased, the content of C_ar3_ and C_ar2_ increased, due to the condensation of PAHs, which demonstrated that excessive bromine could promote the condensation reaction. The amount of CH_ar2_ first decreased slightly and then decreased rapidly as the bromine content increased. This result further revealed that in the presence of a low bromine concentration, the bromination-dehydrobromination process mainly resulted in the oligomerization of aromatic rings through the bridge bonds of their side chains. Meanwhile, too many bridge bonds were formed in the presence of excessive bromine, and these bridge bonds were easily cyclized, aromatized, and even condensed with the aromatic rings. These inferences were also confirmed by the phenomenon of increasing C_ar3_ and C_ar2_ contents.

In general, based on the ^13^C NMR spectra analysis of the isotropic pitch precursors, it was concluded that aromatic groups can be connected through side chains to form bridge bonds under the induction of bromine. In addition to the cross-linking reaction, the condensation reaction can also occur, and the amount of condensation reaction had a positive correlation with that of bromine added into the EO. All of these results were consistent with the TMA analysis. Therefore, effective control of bromine addition is a prerequisite for the controllable preparation of isotropic pitch precursors.

## 4. Conclusions

Bromination-dehydrobromination is a new, effective approach for the preparation of isotropic pitch precursors from the byproducts of coal and petroleum processing. At 110 °C, some of the bromine added to the EO was converted into bromine free radicals, and then the aliphatic side chains of PAHs in EO were substituted by these bromine radicals. When the temperature reached 340 °C, the bromine in the brominated PAHs formed hydrogen bromide with the hydrogen on another PAH, and this hydrogen bromide was removed. Through the removal of hydrogen bromide, the PAHs were cross-linked by side chains, and the isotropic pitch precursors could be controlled. However, the molecules in the EO also underwent condensation reactions at this temperature. As the amount of bromine added increased, the amount of hydrogen on the side chains of the aromatic rings substituted by bromine also increased, which promoted the occurrence of the condensation reaction and increased the condensation degree of molecules in the EO. All of these factors increased the condensation degree of the EO molecules, and the content of PAHs further increased, which improved the aromaticity of the isotropic pitch precursors. Moreover, with the increase in bromine addition, the molecular structure of the isotropic pitch precursors gradually changed from the cross-linked stereo structure to the macromolecular plane structure of condensed PAHs. Therefore, controlling the content of bromine can effectively promote the formation of the cross-linked stereo structure of the isotropic pitch precursors, which provides the feasibility for the subsequent spinning. In the next step, the effect of the cross-linked stereo structure on the mechanical performance of final GPCFs will be studied further.

## Figures and Tables

**Table 1:** Properties of isotropic pitch precursors.

Sample	Basic Pitch	Isotropic Pitch Precursor		Element Content		
	Yield (%)	Yield (%)	*SP (°C)	C (%)	H (%)	C/H
EO-5	67.3	41.8	240	90.81	7.86	11.55
EO-10	70.4	45.2	240	92.06	6.75	13.64
EO-15	71.1	51.1	240	92.81	6.05	15.34
EO-20	68.4	58.4	240	93.85	5.13	18.29

*SP represents the softening point.

**Table 2:** The average molecular weight and molecular compositions of isotropic pitch precursors.

Sample	*AMW	Molecular Composition (%)				
		Small Molecules	Monomer	Dimer	Trimer	Tetramer
**EO-5**	638.04	0.12	6.70	54.37	25.58	12.94
EO-10	667.92	0.13	6.95	49.09	28.41	15.42
EO-15	707.87	0.05	5.25	43.28	32.38	19.04
EO-20	737.24	0.05	5.20	39.87	33.45	21.43

*AMW represents the average molecular weight; small molecules: <202 *m*/*z*; monomer: 202~388 *m*/*z*; dimer: 388~645 *m*/*z*; trimer: 645~890 *m*/*z*; and tetramer: 890~1120 *m*/*z*.

**Table 3:** Carbon atom determination of isotropic pitch precursors by ^13^C NMR spectra.

Sample	Aliphatic Carbon (%)			Aromatic Carbon (%)				fa
	CH_3_^a^	CH_2_^b^	CH_a2_^c^	CH_ar_^d^	C_ar3_^e^	C_sar_^f^	C_ar2_^g^	
**EO-5**	6.88	10.04	14.23	1.94	30.02	7.95	28.94	0.69
EO-10	5.47	10.23	14.22	1.85	30.95	8.73	28.55	0.70
EO-15	5.58	9.21	11.69	1.76	33.52	7.42	30.82	0.74
EO-20	5.01	9.43	10.23	1.59	35.42	6.58	31.74	0.75

Note: ^a^ Methyl carbon, 17–23 ppm; ^b^ methylene carbon in the side aliphatic chain, 23–34 ppm; ^c^ Bridge/hydroaromatic structure (methylene carbon in a position to two aromatic rings), 34–50 ppm; ^d^ protonated aromatic carbon, 108–115 ppm; ^e^ peri-condensed aromatic carbon, 115–129.5 ppm; ^f^ aromatic carbons joined to an aliphatic chain, 129.5–132.5 ppm; ^g^ cata-condensed aromatic carbon with both heteroatomic or aromatic substituents, 132.5–160 ppm.
